# Reproductive Behavior and Basic Biology of the Oriental Bamboo-Inhabiting *Anoplomus rufipes* and a Comparison with Frugivorous Dacinae Fruit Flies

**DOI:** 10.3390/insects6040869

**Published:** 2015-10-23

**Authors:** Damir Kovac

**Affiliations:** Senckenberg Research Institute, Entomology I, Senckenberganlage 25, Frankfurt am Main 60325, Germany; E-Mail: damir.kovac@senckenberg.de

**Keywords:** courtship behavior, pheromone calling, lek formation, host plant, life cycle

## Abstract

The reproductive behaviors and mating systems of the fruit-infesting species of the Dacinae tribes Ceratitidini and Dacini are increasingly well understood, while in the non-frugivorous tribe Gastrozonini, data are lacking. In the present study, the reproductive behavior of *Anoplomus rufipes* from North Thailand was studied in the field, other behaviors also in the laboratory. *A. rufipes* mated on young bamboo plants growing in areas destroyed by fire. Exudates of extrafloral nectaries produced by the young bamboo plants provided food for the females. Factors affecting the choice of the mating site were favorable microclimatic conditions and food. Courtship behavior was performed on the upper sides of bamboo leaves and included pheromone calling (abdominal elevation, anal pouch eversion, abdominal pleural distention), anal dabbing, looping flights and a specific lofting/body swaying behavior. The males searched individually for females or formed leks containing up to four males. The reproductive behaviors and lek formation of *A. rufipes* are compared to other Dacinae (*Ceratitis*, *Bactrocera*), and their functions are discussed. Hitherto unknown data on the general biology of *A. rufipes* are also included. *A. rufipes* larvae infested living bamboo shoots of *Cephalostachyum pergracile*, and the observed behaviors of the adults included locomotion, grooming, feeding, oral droplet deposition, bubbling and agonistic behavior.

## 1. Introduction

The reproductive behaviors of fruit flies (Tephritidae) are extraordinary diverse, ranging from resource-based mating systems in which males wait for females at their egg-laying sites and engage in elaborate pushing contests with opponents, to mating systems away from resources in which males perform complex courtship behaviors or offer nutritional “nuptial gifts” to the females [1,2,3,4,5].

The most complex signaling systems in tephritids are found in polyphagous frugivorous tropical species. They include important fruit pests, such as *Anastrepha* (Neotropical; subfamily Trypetinae) or *Ceratitis* and *Bactrocera* (Old World; subfamily Dacinae) [6]. Mating occurs on fruits or leaves of the host plant, and the courtship behavior involves signaling with pheromones or acoustic and visual displays (“calling”). A striking phenomenon among leaf-calling species is the formation of male mating aggregations (“leks”) that are visited by receptive females.

The subfamily Dacinae species of Ceratitidini (for example, the notorious medfly *Ceratitis capitata* (Wiedemann)) or Dacini (for example, *Bactrocera dorsalis* (Hendel), *B. cucurbitae* (Coquillett)) mostly develop in fruits belonging to a wide spectrum of families of plants. Species belonging to the third tribe of Dacinae, Gastrozonini, are non-frugivorous, and all species with known host plants develop in bamboos (Oriental species) or in other grasses (Afrotropical species) [7,8,9,10].

Many investigations have addressed the reproductive behaviors of fruit-infesting Dacinae of economic importance, whereas observations on the reproductive behaviors of Gastrozonini are rare. Some Gastrozonini species were occasionally found to copulate on cut bamboo shoots [10], but complex reproductive behaviors have not been observed.

The present study deals with *Anoplomus rufipes* Hardy 1973 and provides the first description of complex reproductive behavior and lek formation in Gastrozonini. Hitherto unknown data on the host plant and general biology of *A. rufipes* are included, since the ecology of a species shapes its communication and mating system. The reproductive and lek-forming behavior of *A. rufipes* are compared to other Dacinae, and their functions are discussed.

## 2. Experimental Section

### 2.1. Location of the Study Area and Climate

Field observations of *Anoplomus rufipes* were conducted in NW Thailand, Province Mae Hong Son, district Pangmapha. The mountainous Pangmapha district is primarily covered with dipterocarp monsoon forests. Bamboo stands are common in areas affected by farming. The seasons occurring in northern Mae Hong Son include the dry season (February until April), the rainy season (April/ May until October, the highest amounts of rainfall occurring in July and August) and the cool season (November until February).

### 2.2. Sampling Methods

The *A. rufipes* adults were collected at altitudes between 600 and 900 m by using a sweeping net. Collecting was done between 2004 and 2015 at irregular intervals during all months of the year, except January. Larvae were collected in 2003, 2008 and 2014 by cutting off internodes infested by *A. rufipes* and transporting them in plastic bags to the laboratory.

### 2.3. Field Observations

**Figure 1:**
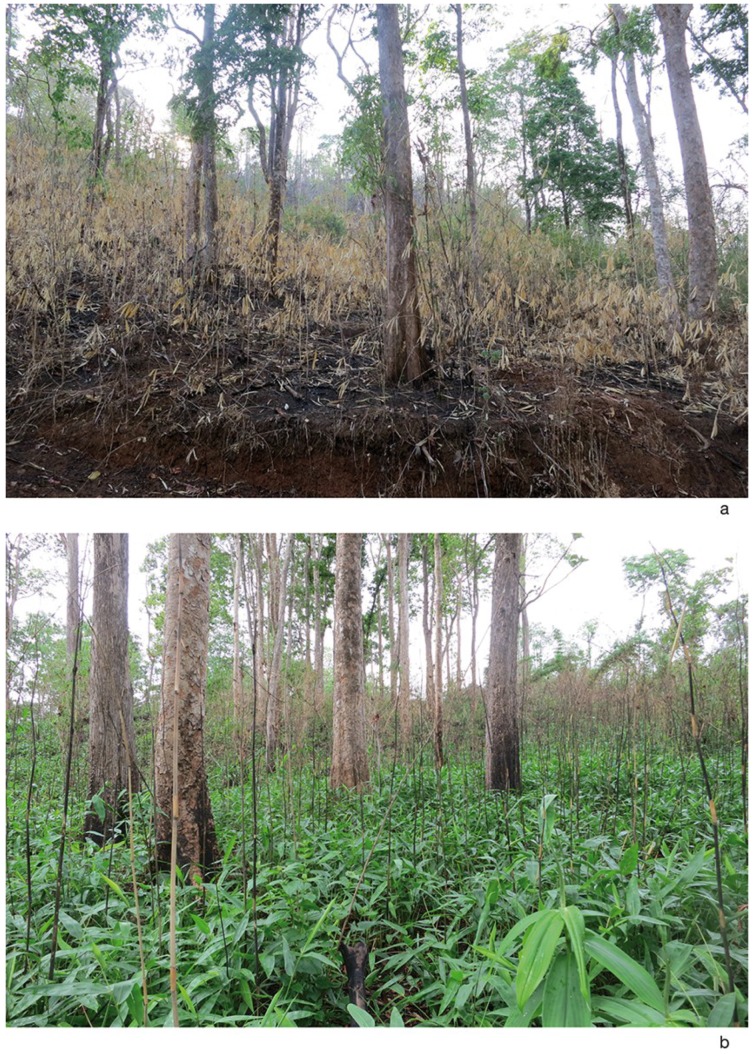
Mating sites of *A. rufipes*. (**a**) On 24 April 2014, about 5 weeks before the first mating was observed, the bamboo undergrowth in the study area was destroyed by fire, and *A. rufipes* was not present. (**b**) On 29 May 2014, at the beginning of the rainy season, young bamboo plants growing from rhizomes of *Pseudoxytenanthera albociliata* were already about 50 cm tall, and *A. rufipes* was observed to mate on the upper surfaces of bamboo leaves. Note the burnt thin bamboo shoots sticking up from the ground.

Field observations of the reproductive behavior of *A. rufipes* were conducted at a site near the village of Soppong. The study area was about 300 m long and 100 m wide and was covered with light tree vegetation and bamboo undergrowth. The area was delimited by the main road and abandoned fields. The dominant bamboo species in the area was *Pseudoxytenanthera albociliata* (Munro), while *Cephalostachyum pergracile* Munro and *Dendrocalamus strictus* (Roxburgh) Nees were also present. At the beginning of the observations, about half of the area was destroyed by fire (Figure 1a). Later on, young bamboo plants started to grow from rhizomes in burnt areas.

An area of *ca*. 25 × 25 m^2^ was chosen as the main study site, because most leks were found in this location. This area was located in the middle of the burned area under several shady trees (Figure 1b), which were surrounded by an unshaded bamboo undergrowth strip. The bamboo species growing in and around the 25 × 25 m^2^ area was *P. albociliata*. In two larger neighboring areas containing young bamboo plants growing on burnt ground, the males were usually searching individually for females. The observations on reproductive behaviour were conducted on 21 days between 26 May and 22 June 2014, usually between 11:00 and 17:00, on three days between 8:00 and dawn. Pheromone-emitting (calling) males were searched for by walking clockwise around the main study site along a rectangular path. Two additional pathways crossed the central part of the main study site.

### 2.4. Rearing

In the laboratory, internodes infested by *A. rufipes* were kept in plastic containers provided with moist tissue (toilet paper) placed on the bottom. Mature larvae leaving the internode were transferred to smaller containers. The larvae pupariated between the moistened tissue. After emergence from puparia, the adults were transferred to glass terraria (30 × 20 × 20 cm^3^) containing moistened tissue paper and bamboo branches immersed in water. Drops of water mixed with sugar, honey or yeast were placed on the bamboo leaves as food for the flies.

### 2.5. Behavioral Observations in the Laboratory

Altogether, five males and one female were available for laboratory observations between 16 February and 14 April 2015. The specimens had been reared form larvae collected in November 2014. The males emerged on 16 February (2×), 19 February, 7 March and 23 March 2015 and the female on 1 April 2015. The five males were kept together in two terraria (two and three males per terrarium), while the female was kept individually. Interactions between males and the single female were tested between 9 April and 14 April 2015 by placing the female alternately into a terrarium containing two or three males. The tests were conducted for two hours daily on six consecutive days.

## 3. Results

### 3.1. Habitat and Temporal Occurrence of the Adults

Field-observed adults were recorded between 6 March and 29 October (combined data for the years 2004 to 2014). In March through to mid-May, *i.e.*, during the hot season, the adults gathered on herbaceous (usually non-bamboo) vegetation growing along small, shady mountain streams. After the rains had started, the adults dispersed to surrounding areas and remained on broad-leaved bamboo species growing in the secondary forest or at the edges of fields. On one occasion (3 July 2004), a larger number of *A. rufipes* males and females were seen on the leaves of the large grass *Thysanolaena latifolia* (Roxb. Ex Hornem.) Honda.

### 3.2. Host Plant and Larval Development

**Figure 2:**
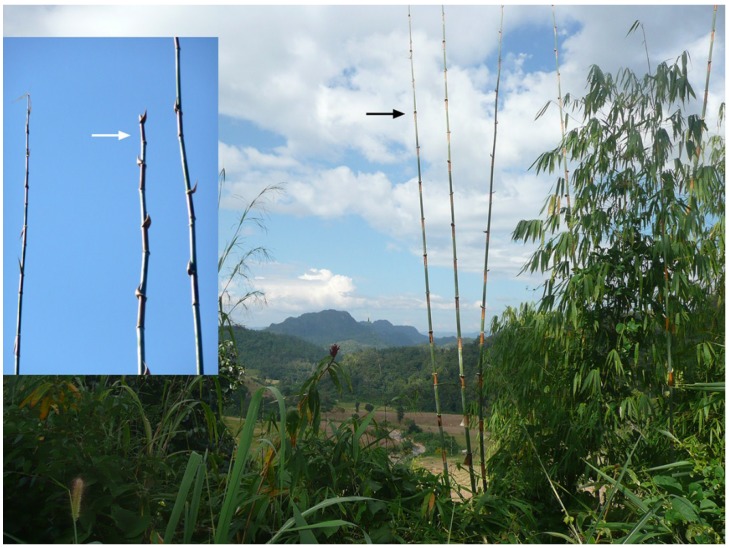
Habitat of *A. rufipes* in November (N Thailand, Pangmapha district). The black arrow indicates a bamboo shoot of *Cephalostachyum pergracile*, the host plant of *A. rufipes*; Inset: *A. rufipes* larvae inhabit the fifth to sixth internode below the apex of a bamboo shoot. After some time, the apical four to five internodes die off and fall to the ground, whereas the infested internode (white arrow) remains on the top of the shoot for a longer time.

The larvae of *A. rufipes* were discovered in the shoots of the bamboo *Cephalostachyum pergracile* (Figure 2) and in one case in a shoot of *Pseudoxytenanthera albociliata*. Infested shoots of *C. pergracile* were 2 to 10 m tall. *A. rufipes* larvae inhabited only a single internode of an infested bamboo shoot. Altogether, fifteen *C. pergracile* internodes containing *A. rufipes* larvae were collected between 6 and 27 November, in 2008 and 2014. *A. rufipes* larvae found in *P. albociliata* were already dead (single internode found on 28 October 2013).

The internode infested by *A. rufipes* was the fifth to sixth internode below the apex of a bamboo shoot. Due to the feeding activities of the larvae, the infested internode, as well as the four to five apical internodes died off and turned yellow. After some time, the apical part of the shoot fell to the ground, whereas the infested internode remained attached to the shoot (Figure 2, inset). It was held in place by the culm sheath tightly enclosing the lower half of the internode. After larvae had left, branches started to grow from the bud located at the base of the infested internode, and this internode also fell down to the ground.

**Figure 3:**
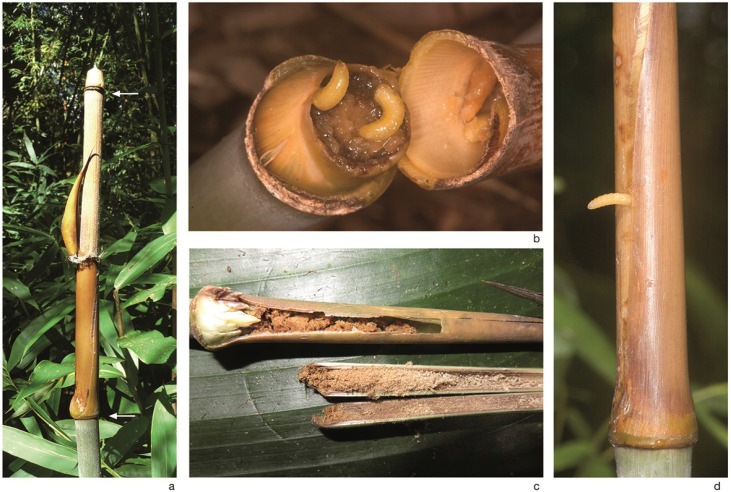
Larval habitat and feeding traces of *A. rufipes*. (**a**) Infested internode of *C. pergracile*, which was located on the top of a bamboo shoot, *ca*. 8 m above the ground. The lower half of the internode, where the larvae feed, is enclosed by the stiff and tight culm sheath. White arrows indicate the upper and lower end of the internode; (**b**) Larvae of *A. rufipes* (culm sheath broken off); (**c**) Feeding traces of *A. rufipes* in a small internode. The basal part of the internode wall was entirely devoured (internode pulled out, sheath and internode cut open). Note the yellow-green brunch bud at the base of the internode (left); (**d**) *A. rufipes* larva leaving the internode by squeezing its way between the overlapping culm sheath.

Internodes inhabited by *A. rufipes* (Figure 2 and Figure 3a) were 7.6 to 24 cm long and 7 to 20 mm wide (diameter measured in the middle of the internode, *n* = 14). The internodes were hollow or sometimes contained a pith-like tissue. The yellow-colored larvae of *A. rufipes* (Figure 3b) fed both on the pith-like tissue and on the internode wall. The basal 2 to 4 cm of the internode wall, which were softer than the upper part of the internode, were entirely devoured (Figure 3c). In total, fifteen internodes containing *Anoplomus* larvae or showing their feeding traces were found. The infested internodes contained up to 40 larvae. In some internodes, there were only a few larvae, because judging from the feeding traces, most larvae had already left. Mature larvae abandoned the internode by squeezing themselves between the overlapping culm sheath (Figure 3d) and then skipping to the ground. In two cases, larvae got stuck with their rear ends between the overlapping culm sheath and did not manage to leave the internode even after several days. Despite the damaged internode wall, the larvae remained protected from the outside world by the culm sheath. However, sometimes, the culm sheaths were pecked open, probably by predatory birds.

Seventeen larvae collected in November 2008 and 2014 were successfully reared to the adult stage. In the laboratory, mature larvae abandoned their internodes in November to about mid-December. They created small chambers in the moist paper placed on the bottom of the rearing container. The larvae remained in their chambers in a curved position for up to four months prior to pupariation. One male emerged 29 days and one female 28 days after pupariation, *i.e.*, the puparial stage lasted about one month. The seventeen adults reared in the laboratory emerged from the puparia between 11 February and 11 May of the following year.

### 3.3. General Behavior of the Adults

#### 3.3.1. Locomotion and Wing Displays

*Anoplomus rufipes* individuals were active during the daytime. They were good fliers and could probably cover a distance of many kilometers per day. When at rest, *A. rufipes* usually stayed on the upper sides of bamboo leaves and showed the behavioral patterns compiled in Table 1.

**Table 1:** Compilation of behavioral patterns of *A. rufipes* adults observed during the present study in the laboratory or in the field. Most field observations were conducted during the courtship period in May/June 2014, while the laboratory observations were conducted in February until April 2015 using newly-emerged adults. Courtship behavior was not observed in the laboratory, indicating that mating does not occur immediately after the emergence of the adults. In the laboratory, the lofting/body swaying behavior was employed as a threatening gesture, whereas during the courtship period in the field, it was observed only in the context of courtship. * The enantion and supination movements were more pronounced and faster during courtship.

Behavioral Patterns	Behavioral Context	Laboratory	Field
Walking, flying	Locomotion	x	x
Turning towards moving objects	Orientation	x	x
Enantion (wing display)	Function?	x	x *
Supination (wing display)	Function?	x	x *
Lunging forwards	Agonistic behavior	x	x
Pounding with forelegs	Agonistic behavior	x	x
Lofting (wing display)	Agonistic behavior	x	-
Lofting/ body swaying	Agonistic behavior	x	-
Wing vibration	Agonistic behavior	x	-
Grooming movements	Cleaning behavior	x	x
Dabbing with proboscis	Feeding, excretion behavior	x	x
Oral droplet deposition	Feeding, excretion behavior	x	-
Oral droplet extrusion	Feeding, excretion behavior	x	x
Feces disposal (+ abdominal dragging)	Feeding, excretion behavior	x	x
Abdominal elevation (calling)	Courtship behavior	-	x
Anal pouch eversion (calling)	Courtship behavior	-	x
Abdominal pleural distention (calling)	Courtship behavior	-	x
Lofting/body swaying	Courtship behavior	-	x
Looping flights	Courtship behavior	-	x
Anal dabbing	Courtship (marking) behavior	-	x

The wings of *A. rufipes* males were always parted and roughly held in two positions. In the basal position, the wings were slightly parted (angle of costal margin to the midline of the body *ca*. 40°), and from time to time, the flies moved them quickly forward and backward (enantion; see Figure 11 in [11]). In the second position, the wings were held *ca*. perpendicular to the midline of the body; the ventral side of the wing was turned more or less toward the front, and the wings were sometimes quickly moved backward and forward (supination; see Figure 14 in [11]). Both during enantion and supination, the wing movements were synchronous or slightly asynchronous. The wings were moved only for a short distance (at a 20 to 30-degree angle) and about once in 5 to 10 s. If the flies were aroused, the movements were more intense and occurred more often (Table 1). The females held their wings about perpendicular to the midline of the body most of the time. Additional types of wing display were observed during aggressive interactions and mating (see below).

In the terrarium, the flies mostly stayed upside down on the glass ceiling and showed the same wing displays as in the field.

#### 3.3.2. Grooming

Grooming was frequently observed, and the behavioral patterns were identical both in the lab and in the field. The grooming patterns were as follows: The head was turned sideways and the distal parts of the fore tibiae cleaned simultaneously the vertex, the eyes, the mouthparts and the antennae. Between and at the end of these grooming sequences, the fore tarsi and tibiae were rubbed together. The body was cleaned by the hind tibiae. They rubbed along the dorsum from the frontal part of the thorax towards the tip of the abdomen. The wings were lifted to allow cleaning of the dorsum. Following body cleaning, the hind tarsi and tibiae were rubbed together.

The wings were cleaned in two ways. The first method involved raising both hind legs and rubbing simultaneously along the dorsal part of the wings from the wing base towards the apex. The second method involved two hind legs grooming one wing. The right wing was cleaned by the right hind leg on top and the left hind leg on bottom and *vice versa*.

Each leg was groomed by two other legs. The left front leg was cleaned by the right front leg and the left middle leg and the right front leg by the left front leg and right middle leg. Each middle leg was cleaned by both forelegs. The left hind leg was cleaned by the right hind leg and left middle leg and the right hind leg by the left hind leg and the right middle leg.

#### 3.3.3. Feeding and Feces Elimination

Field-observed *A. rufipes* males and females dabbed their labella onto leaf surfaces, extrafloral nectaries, bird droppings, plant juices oozing from injured bamboo shoots, plant juices sticking to the sweeping net, human urine adhering to leaves or tree trunks and sweat located on the hand of the investigator, on a backpack or other pieces of equipment. The flies also imbibed rain or dew droplets from leaves.

**Figure 4:**
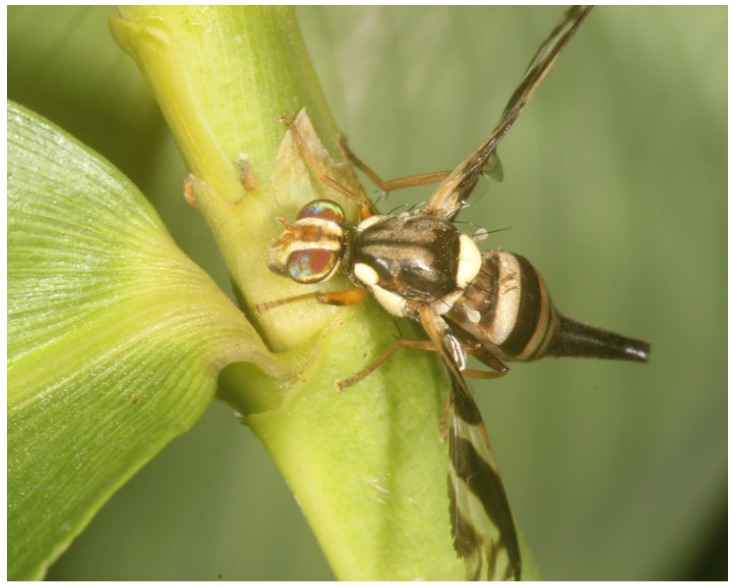
*A. rufipes* female feeding on secretions of a bamboo extrafloral nectary of *Pseudoxytenanthera albociliata*.

In May and June 2014, during the observations on reproductive behavior, females were often seen to feed on exudates oozing from extra-floral bamboo nectaries of *Pseudoxytenanthera albociliata*. Bamboo nectaries were situated on the auricles (Figure 4), which were located at the base of the leaf-like part of the culm sheath (=blade; see [12]). The nectaries were functional on freshly-emerged bamboo leaves and ceased to produce secretions after a while, *i.e.*, functional nectaries were only present in the apical part of a growing bamboo branch. The nectaries were regularly visited by ants and also exploited by other insects, for example *Bactrocera* sp. or other flies, as well as beetles and cockroaches. When an ant approached an extrafloral nectary, the *A. rufipes* female backed away or flew to a nearby location at a distance of 2 to 3 cm. The female returned to the extrafloral nectary immediately after the ant had left. If other flies approached, they were evicted by the *A. rufipes* females. The females remained on or in the vicinity of a single extra-floral nectary for many hours.

Laboratory-observed males of *A. rufipes* produced large, clear fecal droplets at the tip of their abdomen and placed the droplets on the substrate by lowering their abdomen. A field-observed female staying on the upper side of a leaf elevated her abdomen and exuded a drop of a clear liquid (ovipositor slightly extruded). Subsequently, she bent her abdomen downwards and dragged it behind her for about 6 cm.

#### 3.3.4. Oral Droplet Deposition and Oral Droplet Extrusion (Bubbling)

**Figure 5:**
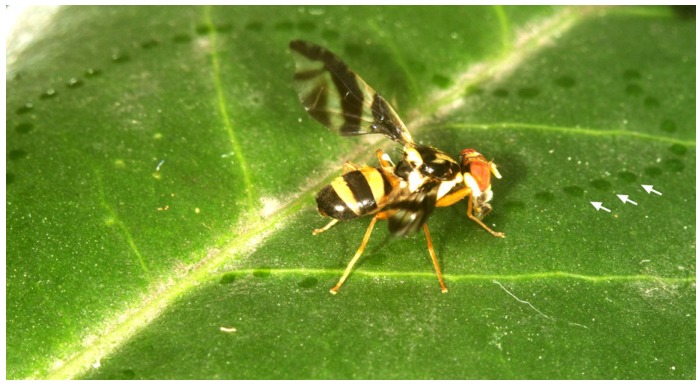
Deposition and reingestion of oral droplets (white arrows) by an *A. rufipes* male.

A frequent behavior observed in the lab was regurgitation and deposition of transparent liquid drops onto leaf surfaces and reingestion of the remaining dry solids once the droplet had dried (Figure 5). The flies walked on the leaf surface in curving lines and, after each step, deposited a flat droplet with their proboscis (one to two droplets per second). They placed up to 24 droplets in a row and then paused, groomed or produced new droplet lines nearby. The diameter of the droplets was 0.66 mm, and the distance between them was 0.13 mm or longer. After evaporation, the droplet location was still clearly visible and often shiny. After some time, the flies stopped producing droplets and started to collect the more or less dry solids. They took up the solids one by one with a single dab of the proboscis. At the end of the droplet line, they searched for further droplet lines until the last droplet was ingested. After droplet deposition, the flies usually groomed.

In one case, a male had deposited 115 droplets with a diameter of 0.66 mm, *i.e.*, the total surface of all droplets was 3.9 cm^2^. Sometimes, the flies started to deposit and reingest droplets for a second or a third time, and the whole procedure lasted more than one hour. When males were disturbed, they started to collect the droplets faster than usual.

The flies usually collected the droplets shortly after they had been deposited. However, in one case, the droplets did not evaporate due to the high humidity in the terrarium. The male checked the droplets from time to time, but did not collect them. The male reingested the drops only during the next day, after humidity had decreased.

**Figure 6:**
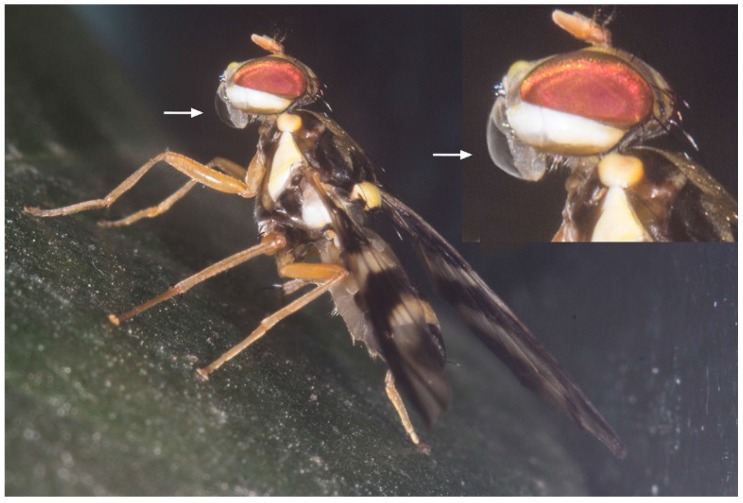
Bubbling behavior in an *A. rufipes* male. A droplet is extruded at the tip of the proboscis (arrow). The inset shows the enlarged head.

Another common behavior involving oral droplets was “bubbling”. Both males and females exhibited fast pumping movements of their proboscis in the lab, as well as in the field. Their proboscis was protruded and contracted about two times per second or sometimes slowly stretched out. Subsequently, a clear liquid droplet appeared at the tip of the proboscis (Figure 6). The droplet was flat or roundish and sometimes only visible due to the reflection of the light at the water/air interface. The fly remained motionless for 10 to 68 s (M = 38 s, *n* = 19). Subsequently, the droplet was retracted, the pumping recommenced and a new droplet extruded. The bubbling behavior lasted 20 min or longer and was often accompanied by grooming.

#### 3.3.5. Agonistic Behavior in Males and Females

**Figure 7:**
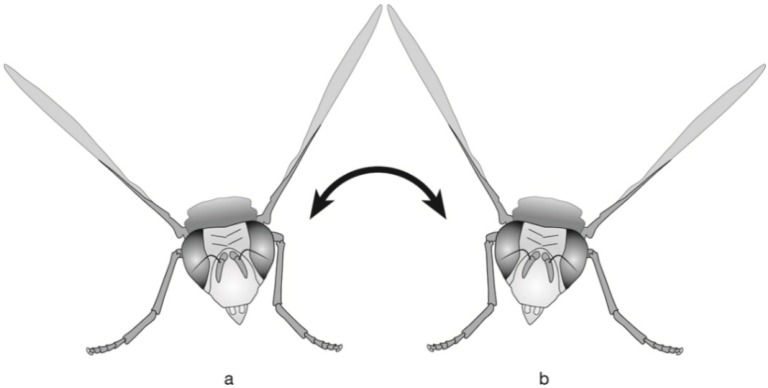
Illustration of the lofting/body swaying behavior of *A. rufipes* males. The wings are raised and arrested at an angle of about 45°, and the body is tilted alternately to the sides, while approaching the opponent (agonistic behavior) or mate (courtship behavior).

Males kept in a terrarium usually walked on the ceiling and often approached each other. As soon as the distance between the males was less than 1 to 2 cm, they showed the following aggressive behaviors: (1) the attacking male lunged forwards, sometimes lifting his forelegs in order to push the opponent away; (2) the attacking male lifted and lowered his wings one to four times while facing or approaching the opponent; the wings were lifted from the basal wing position to the front, until the wings were extended upward; this wing movement was described by Headrick and Goeden [11] as “lofting” (see Figure 13 in [11]); (3) the attacking male lofted his wings, arrested them in the raised position and alternately swayed the body to the right and left up to six times, while approaching the opponent; this combined movement is termed lofting/body swaying in the present study (Figure 7). Unlike the body swaying described by Headrick and Goeden [11] for Tephritinae (“moving the body side-to-side over the legs in a plane parallel to the substrate), it was rather a tilting of the whole body to the sides. In Tephritinae, the tarsi remained stationary during swaying, while in *A. rufipes*, the male was usually approaching the opponent. Finally, an additional behavior was observed, albeit two times, that is interpreted as aggressive behavior: a male vibrated with his wings when facing another male.

Heavy fighting was observed between a male depositing droplets and an intruding male. The intruder alighted on the leaf, and both males lunged forward and turned around each other several times. Subsequently, the intruder was evicted and remained on the glass wall, about two cm beside the leaf. The resident male continued to deposit or to collect droplets, but he paused from time to time, walked to the leaf edge towards the opponent and repeatedly raised his wing. After a while, the intruder attacked the resident male again and pushed him repeatedly with his forelegs. The attacked male ducked down and hardly defended himself, but did not retreat either. After several minutes of constant attacks, the intruder retreated.

Interactions between males and one female were observed on six consecutive days in the laboratory (see the Experimental Section). Courtship behavior was not observed. Males evicted the female by lunging towards her, lifting and lowering their wings or performing the lofting/body-swaying behavior. Occasionally, the female evicted smaller males by lunging towards them or briefly moving her wings forwards and backwards. In the field, female aggressive behavior was observed only towards other fly species, while feeding on extrafloral nectaries.

### 3.4. Reproductive Behavior

#### 3.4.1. Mating Site and Temporal Occurrence of the Courtship Behavior

Sporadic observations at the mating site began on 20 April 2014, about five weeks before the first *A. rufipes* mating was detected. The vegetation consisted of a light forest and bamboo undergrowth. At the beginning of the observations, about half of the area was destroyed by fire, and dead culms were sticking up from the bare ground (Figure 1a). In areas not destroyed by fires, the bamboo stems were about 2 to 5 m high. Due to the absence of rain for about six months the vegetation was dry and the bamboo foliage was sparse and partly yellow. Only bamboos growing beside a pond located in a depression of a dry streambed were relatively green and provided some shade.

On 20 April 2014 and on the following days, *A. rufipes* specimens were swept from bamboo branches growing beside the pond, but not from the bamboos located in dry areas. After the rainfalls had started at the end of April and the beginning of May, young bamboo plants (branches with leaves, not the typical bamboo shoots) started to grow from rhizomes located in burned areas forming small bushes. The small bamboo bushes were regularly inspected for tephritids, and on 24 May, several *A. rufipes* specimens were observed to walk and rest on the upper sides of leaves.

The first *A. rufipes* mating was observed on 26 May 2014 at 15:00. During the following three weeks, *A. rufipes* males were seen to perform pheromone calling on every observation day. *Anoplomus* individuals preferred to stay in shady areas below the trees. They avoided areas exposed to sun, where the leaves of the young bamboo bushes curled up during midday heat. When weather became increasingly cloudy and rainy, the *A. rufipes* specimens spread to open bamboo areas, as well. During the mating period, *A. rufipes* specimens stayed on the fast-growing bamboo bushes and were not detected on branches of older, tall bamboo plants. At the beginning of the mating observations (26 May 2014), the young bamboo bushes were about 20 to 40 cm tall. At the end of the observational period (22 June 2014), they had reached a height of about 1.60 to 1.80 m.

The males were observed to call from 26 May until 16 June 2014. On 19 and 21 June, sporadic males were present at the study site, but calling behavior was not observed. On 22 June 2014 (the last day of the field observations), *A. rufipes* was not detected in the study area.

In April/July 2015, observations were carried out at the same study site as in 2014. The vegetation was not affected by fire as in 2014, and *A. rufipes* was not detected. Besides the matings seen in 2014, there was one additional observation of an *A. rufipes* pair copulating on a bamboo leaf on 15 October 2007.

#### 3.4.2. Aggregation Phase

##### Searching for Mates and Pheromone Calling

**Figure 8:**
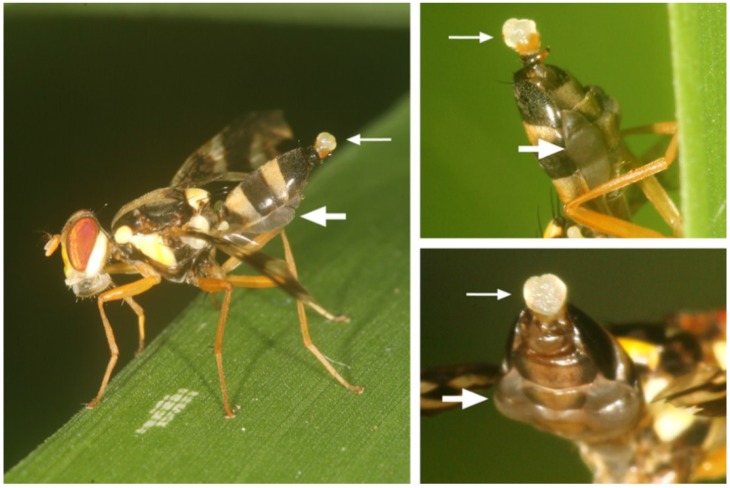
(Left) Typical pheromone calling posture of *A. rufipes* at the edge of a bamboo leaf. The male is looking downwards; the abdomen is raised; a white pouch (evaginated rectum) is visible at the tip of the abdomen (upper thin arrow); and the lateral parts of the abdomen are distended (lower thick arrow). (Upper right) Enlarged abdomen seen from the ventro-lateral position. (Lower right) Enlarged abdomen seen from the ventro-caudal position.

During the courtship period, the males were permanently searching for females on the upper side of bamboo leaves and performing pheromone calling. During pheromone calling, the abdomen was elevated; a whitish inflated pouch (everted glandular anal membranes) was visible at the tip of the abdomen, and the abdominal pleurae were inflated (Figure 8). Pheromone calling occurred mainly in the afternoon, from noon until about 17:30. During that time, the abdomen was permanently kept in the calling position, even when grooming or feeding on bird droppings. Early in the mornings or in the late afternoon, the males were just walking on leaves or feeding.

Males searching for mates were permanently moving between bamboo leaves and quickly moving their wings. The wing movements (enantion) were faster than in non-sexual situations (one to two wing beats per second), and the range of the movements was larger. The males remained on a leaf for just a few seconds up to two minutes and then proceeded to the next leaf. Longer stays lasting up to 11 min occurred, if males were resting, grooming themselves or feeding. Occasionally, the males also alighted on large leaves belonging to other plants or they rested on the thin stems of bamboo plants for a few seconds.

After arrival on a bamboo leaf, the calling males walked on the leaf surface for a few seconds with a raised abdomen and wings moving quickly forward and backward. Subsequently, they stopped at the lateral edge of the leaf adopting a typical posture (Figure 8): the abdomen with the everted anal pouch remained elevated; the wings were brought forward, and the head was directed slightly downwards, thus appearing to monitor the surroundings. The male remained motionless in this position for a few seconds and then proceeded to the next bamboo leaf.

Occasionally, males calling from the edge of a leaf flew upwards (up to nine cm) or sideways (distance 3 to 4 cm) and then returned to the same spot while performing loops. One looping flight lasted 2 to 3 s and was repeated three or more times in a row.

Calling *A. rufipes* males occasionally touched the leaf surface with the tip of their abdomen for about one second (anal dabbing, also termed anal dipping or anal touching [13,14]). One male performed anal dubbing twice during an observation period of 1.50 h and one male once during an observation of 1.00 h.

##### Agonistic Behaviors during Courtship

While calling from the leaf edges, the males examined their surroundings. As soon as an insect walked or flew at a distance of less than *ca*. 30 cm, the male instantly flew towards the insect, alighted on a neighboring leaf and then proceeded to the leaf occupied by the insect. If the insect turned out to be an opponent, the male lunged at the opponent, elevated the front part of his body by stretching the middle legs and pounded with his forelegs against him. At that stage, one of the opponents usually gave up or sometimes both males simultaneously departed. If the fighting continued, one male tried to climb onto the other. The encounters usually lasted one to two seconds, but sometimes fighting was fierce and lasted several seconds. The males apparently recognized the sex of a conspecific male from a distance of about 1 to 2 cm.

Particularly active males (recognizable by faster movements, looping flights) flew to any object moving within their visual fields (*ca*. 30 cm), and if the object turned out to be a male, they usually dispelled him from his leaf. The attacking males did not return to their previous leaves, *i.e.*, they did not protect a certain leaf territory. If the escaped male remained on a nearby leaf, the winning male attacked him again. If the inferior males escaped to a more distant leaf beyond the visual field of the winning male, he was not bothered again. Males participating in a lek were repeatedly engaged in aggressive interactions with the same males.

##### Lek Formation

Males were calling individually or they gathered in groups (leks) of up to four males. At the main study site, the leks were found at the periphery of the bamboo undergrowth area where bamboo was less dense and mixed with other plants. Leks were observed between *ca*. 13:00 and 17:00.

Males participating in a lek behaved like single males. They performed pheromone calling and constantly changed their locations, *i.e.*, they did not occupy a fixed leaf territory. The distance between the males was up to 1 m and was always changing. Aggressive interactions occurred if the distance became less than *ca*. 30 cm. The lek covered an area of about 3 × 2 m^2^, and its location was slowly shifting. Occasionally, the lek disintegrated and was re-established after some time at a nearby location. Leks were observed during the whole three-week courtship period. One lek was seen at the same corner of the main observation site (area of *ca*. 6 × 6 m^2^) for about two weeks. 

Females were usually not present on the upper surfaces of leaves in the vicinity of leks. They could only be detected if bamboo plants were carefully examined from various directions, because the females usually stayed on bamboo nectaries and were hidden by leaves. After a careful examination of the plants, usually up to four females were found in or near a lek area. However, females were occasionally seen to fly more or less straight-lined from leaf to leaf, to dab the leaf surface, pause at the edge of the leaves and look downwards. This behavior was similar to the mate searching behavior of the males.

#### 3.4.3. Courtship Phase

After detecting a female, the male flew towards her, alighted on a neighboring leaf and then proceeded to the female leaf. Subsequently, the male approached the female with elevated abdomen. At a distance of about 1 to 2 cm, the male performed the “lofting/body swaying” behavior, which was already described in the context of aggressive behavior (Figure 7). Occasionally, the lofting/body swaying behavior was also performed in front of other males or other insects (for example, *Ichthyurus* sp. beetle, a small elongated weevil and a small cockroach), but it was always quickly interrupted, *i.e.*, the wings were just shortly raised, and the body tilted just to one side. If the females remained on the spot, the male flew on the back of the female and initiated copulation.

Frequently, males did not succeed in copulating. In ten cases, the females departed while the male was performing the lofting/body swaying behavior; in one case, the courting male was disturbed by another male; and in three cases, courtship was interrupted by heavy rain. In many other cases, the females did not react to approaching males and just continued to feed on bamboo nectaries, even if the searching males were very near, for example at a distance of 10 cm. If the female was not moving, she was not detected by the male.

In one case, a female was observed to approach a calling male. The female alighted on a neighboring leaf and was immediately detected by the male. The male flew to the same leaf and approached the female showing the normal wing display movements (enantion). Subsequently, the male mounted the female straight away, thus skipping the lofting/body swaying display.

#### 3.4.4. Copulatory Phase

**Figure 9:**
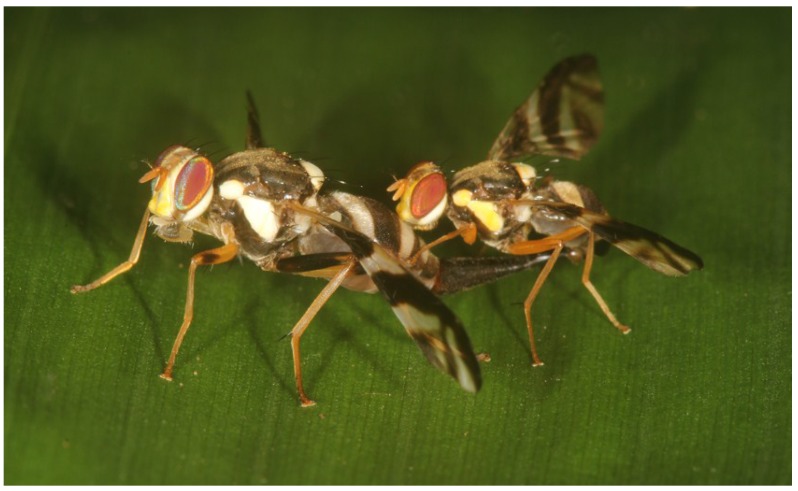
*A. rufipes* couple mating on the upper surface of a bamboo leaf. The forelegs of the smaller, yellowish male rest on the abdomen of the larger black and white female.

Copulation in the field was observed four times (three times at the main study site near a lek area). In two cases, the flies were already copulating when they were detected, and in two cases, mounting was witnessed. During copulation, male’s foretarsi embraced the female abdomen; the head seemed to touch the female abdomen; and the middle and hind legs rested on the surface of the bamboo leaf (Figure 9). A few minutes after mounting, the females started to move around, always connected to the males. The females walked, flew to neighboring leaves, groomed, performed bubbling or fed on bamboo nectaries. During these activities, the males walked simultaneously with the females or were carried along when losing contact to the ground or during flight. Most of the time, the couple remained on the upper side of leaf surfaces, but sometimes, it also rested on the underside of a leaf. In all four cases, the track of the couples was lost between bamboo leaves after 15 to 90 min.

## 4. Discussion

### 4.1. General Biology and Behavior

#### 4.1.1. Host Plant Phenology, Larval Development and Life Cycle

The main host plant of *A. rufipes* in the Pangmapha district was *C. pergracile*. Dead larvae were also found in an internode of a *P. albociliata* shoot, but only in one instance, despite intensive investigations of Gastrozonini inhabiting this bamboo species. The larvae always killed the infested internode and caused the apical part of the bamboo shoot to die off and fall to the ground. The lower part of the bamboo shoot was not damaged, and growth was not affected.

*A. rufipes* colonized the fifth to sixth internode below the apex of a *C. pergracile* shoot. The reason for this phenomenon was probably that the upper internodes were completely covered by the hard internode sheaths. Gastrozonini cannot pierce the hard bamboo culm sheath; they rather deposit their eggs below the edges of internode sheaths [10]. On the other hand, the lower internodes had already started to harden and were not suitable as larval food.

*Ichneumonopsis burmensis*, another Gastrozonini species with known biology, also occupied the fifth to sixth internode below the apex of a bamboo shoot [12]. In this case, the bamboo shoots were always thinner, the internode was occupied by a single larva and the host plant was always *P. albociliata* (falsely identified as *Melocalamus compactiflorus* (Kurz) Bentham in [12]).

Large bamboo shoots of *C. pergracile* (mature bamboo stands) started to grow in August, and occasional small shoots were present in July. In *P. albociliata*, thin bamboo shoots were present in June/July, whereas larger shoots started to grow in August, with most shoots found between September and November. Shoots of other bamboo species appeared at about the same time, for example large shoots of a *Dendrocalamus* sp. started to grow at the beginning of July. Many of the small shoots growing in June/July were small sized (thin) and not suitable for colonization by *A. rufipes*. On the other hand, large (thick) bamboo shoots needed up to two months to reach the suitable size (internode diameter) for colonization. Thus, the maximum time available for colonization of bamboo shoots of any bamboo species in the area was probably less than five months (July to November).

Gastrozonini developing in short-lived, cut bamboo shoots, needed about three to four weeks from oviposition to emergence from the puparium [10]. *Enicoptera gigantea* Enderlein, a species inhabiting living bamboo shoots, needed up to six weeks to reach the adult stage [10]. If it is assumed that the total developmental time of *A. rufipes* is similar to *E. gigantea*, theoretically two or more generations per year would be possible. However, *A. rufipes* larvae were found only in October and November.

In summary, the life cycle of *A. rufipes* appears to be as follows: The adults emerge during the dry season (March to May) and stay on herbaceous plants growing along mountain streams until May/June. At the beginning of the rainy season, *A. rufipes* disperses to surrounding bamboo areas in search for food. Mating starts at the end of May and was also observed on one occasion in October. The eggs are presumably laid in September/October. The larvae develop in October and November. Mature larvae leave the internodes and burrow in the ground. They remain in the larval stage for up to four months, which enables them to change their location, if the environmental conditions become unfavorable.

#### 4.1.2. Food

Tephritidae imbibe liquids or dissolve solid and dry food by regurgitating their crop contents and liquefying them [15]. The adults require moisture for metabolism, sugar (carbohydrates) as a source of energy (flight, courtship activities) and proteins (amino acids), minerals and vitamins as sources for egg-production [16]). Food sources found to be utilized by Dacinae in nature were juices oozing from fruit and associated bacteria (*Ceratitis*, *Bactrocera*), plant leachates suspended in droplets of dew or guttation (*Ceratitis*, *Bactrocera*), bird droppings (*C. capitata*, *Bactrocera oleae* (Rossi)) and extrafloral nectaries (*Bactrocera*) [17,18,19,20].

The water and food sources utilized by *A. rufipes* were basically the same as in other Dacinae. *A. rufipes* imbibed water from dew and rain, and the main sugar sources were probably plant exudates occurring on leaves (leaf leachates), plant wounds and extrafloral nectaries. Proteins (free amino acids) were possibly attained from bacteria living on leaves, bird droppings, urine and sweat. It is remarkable that during the three-week courtship observations, females were feeding on bamboo nectaries most of the time. Exudates of bamboo nectaries provide sugar, but also secrete other substances, such as amino acids [21], and thus, may contribute to the egg production.

#### 4.1.3. General Behavior

*A. rufipes* individuals moved their wings forwards and backwards (enantion), while walking on leaves. Enantion occurred without any apparent stimulus and became more pronounced when the flies were disturbed or during periods of increased activity, for example during courtship. It is not clear whether and to what extent these wing movements are relevant as courtship signals. Enantion typically occurs in tephritid species possessing banded wings, like *Rhagoletis* and *Procecidochares* [11], and not in species with largely hyaline wings, like *Bactrocera*. Headrick and Goeden [11] recognized that some unrelated species having similar wing patterns exhibited similar wing displays. This is also the case in *A. rufipes*. The wing movements and other behaviors (looping flights during courtship) are more similar to *Anastrepha* then to *Ceratitis* or *Bactrocera*.

*A. rufipes*, especially males, usually walk on the upper surface of leaves. This is unusual, because other Dacinae (*Ceratitis*, *Bactrocera* most Gastrozonini) stay on the underside of leaves. Since *A. rufipes* individuals are highly exposed on the upper side of leaves, it is probable that they are protected against predators in some way. One possible explanation could be that they are to some extent mimicking Hymenoptera, since some of the markings seen in *A. rufipes* also occur on wasps, for example the black and yellow-banded abdomen or yellow scutellum on dark scutum. Some Tephritidae are also known to use their wing movements in interspecific communication. When seen from behind, the wing bands of *Rhagoletis zephyria* Snow and *Zonosemata vittigera* (Coquillet) create the illusion of a salticid spider seen face-on, and the resemblance deters other salticids from attacks [22,23].

Some behaviors observed in *A. rufipes*, for example the stereotyped grooming sequences, were performed in exactly the same manner as was described in other tephritids [11]. The bubbling behavior or the oral droplet deposition are maybe also part of the general behavioral repertoire of tephritids. Oral droplet extrusion (bubbling) behavior is widespread in frugivorous and non-frugivorous Tephritidae and was also observed in *Ceratitis* [11,24,25]. Oral droplet deposition was observed in several species of *Anastrepha* [14], in *Rhagoletis* [25] and in *Bactrocera* [26]. Bubbling behavior and oral droplet deposition probably serve to eliminate excess water from the crop by evaporation [24,25].

Tephritid males display aggressive behavior while maintaining a territory, during courtship or sometimes while temporarily protecting feeding sites, but in other situations, aggression is not common [11]. For example, males and females of *Bactrocera flavoverticalis* Drew and Romig were not aggressive towards each other while feeding on sap oozing from fruits of *Strychnos nux-blanda* A.W. Hill (Loganiaceae) in the daytime, but they abruptly started to fight and court the females at dusk [27]). In contrast, *A. rufipes* males were fighting at all times, whenever they came close to each other.

### 4.2. Reproductive Behavior

#### 4.2.1. Reproductive Behaviors in Dacinae

##### Aggregation Phase

During the aggregation phase, *A. rufipes* males elevated their abdomen (“abdominal elevation”), everted their anal membranes (“anal pouch eversion”) and inflated their pleural abdominal pleura (“abdominal pleural distention”) (Table 2). Furthermore, the males touched the leaf surface with the tip of their abdomen, presumably depositing pheromones on the leaf surface (“anal dabbing”).

**Table 2:** Sexual behaviors observed in males of *Ceratitis capitata* (Ceratitidini), *Anoplomus rufipes* (Gastrozonini) and *Bactrocera* spp. (Dacini). Information stems from sources cited in the text. Abbreviations for the different types of signals: P = pheromone, S = sound, V = visual stimulus, T = tactile stimulus.

Behavioral Phases	Male Behavior	*C. capitata*	*A. rufipes*	*Bactrocera*
Aggregation phase (long range)	Abdominal elevation	x	x	-
	Anal pouch eversion (P)	x	x	-
	Abd. pleural distention (P)	x	x	-
	Anal dabbing (P)	x	x	-
	Anus beating/fanning (P/S)	-	-	x/-
	Wing displays (V)	-	x	-
	Looping flights (V)	-	x	-
	Lek formation	x	x	x/-
Courtship phase (short range)	Abdominal elevation	x	x	-
	Anal pouch eversion (P)	x	x	-
	Abd. pleural distention (P)	x	x	-
	Anus beating/fanning (P/S))	-	-	x/-
	Continuous wing vibr. (P/S)	x	-	-
	Other types of stridulation (S)	x	-	x/-
	Head rocking (V)	x	-	-
	Wing display (V)	-	x	-
	Lofting/Body swaying (V)	-	x	-
	Arista tapping (T)	x	-	-
Copulatory phase (contact range)	Wing buzzing (S/T)	x	-	x
	Body rocking (T)	x	-	x
	Surstyli nipping (T)	x	-	-
	Aculeus raising (T)	x	-	-
	Hind tarsi rubbing (T)	x	-	x

*C. capitata* males behaved like *A. rufipes* and performed elevation of the abdomen, anal membrane eversion, abdominal pleural distention and anal dabbing [28]. Both the anal membranes and the pleural pouches are thought to emit pheromones. In *C. capitata*, pheromones are produced in the anal glands that open externally on the last abdominal segment, in pleural glands and also in salivary glands, while in *Bactrocera tryoni* (Froggatt), *B. dorsalis* and *B.* *cucurbitae*, they are produced in their rectal diverticula [29,30,31,32,33,34].

*Bactrocera* is different from *A. rufipes* and *C. capitata*, because the abdominal pleural distention and anal dabbing are lacking. However, pheromone calling behavior is present, and in many species, a unique pheromone distribution (“anal beating”) is found: the calling male wipes off sex pheromone droplets from the anus with the tarsus of his hind leg. Subsequently, the pheromone droplets are transferred to the cubital cell hairs on the vibrating wings. The vibrating wings spray the pheromone to the front, rear and upper sides of the male. The anal beating behavior often occurs at dusk and was described in detail by Kuba and Sokei [35]. During pheromone spraying, the males of *B. tryoni* and other species also emit an audible calling sound that is produced by wings striking along two rows of bristles (pecten) on the third abdominal tergite [36,37]. Anal beating combined with fanning was reported from *B. tryoni* [29,38], *B. cucurbitae* [35,39], *B. dorsalis* [40], *B. neohumeralis* (Hardy) [41], *B. carambolae* (Drew and Hancock) [42] and *B. cacuminata* (Hering) [43]. In some *Bactrocera* species, for example *B. oleae*, anal beating/fanning is not performed, but pheromones are emitted during courtship, as well [44].

During the aggregation phase, visual displays only occurred in *A. rufipes* (enantion and supination referred to as “wing display” and “looping flights”).

##### Courtship Phase

The courtship phase starts when males detect females and perform displays leading to acceptance or rejection of a male by a female. During the courtship phase, *A. rufipes* males continued to perform pheromone calling, but when they came close to the female (*ca*. 1 to 2 cm), they exhibited a specific visual courtship behavior, which is termed “lofting/body swaying” in the present study. During lofting/body swaying, the wings were raised and pointed upwards, while the body was swayed sideways.

In *C. capitata*, the behavior also changed when the male detected a female. The male deflected his abdomen ventrally and vibrated his wings, probably fanning the pheromone toward the female [45]. This fanning behavior was termed “continuous wing vibration” (overview [28]). During the continuous wing vibration, *Ceratitis* produced a characteristic sound. Later on, the males produced a second type of sound (“intermittent wing buzzing”) by rapidly vibrating wings that were moved rhythmically forward and back [28] (in Table 2, referred to as “other types of stridulation”).

During intermittent wing buzzing, *Ceratitis* performed rapid movements of the head (“head rocking”). The sexually dimorphic anterior orbital bristles of the head or the reflective white area of pubescence on the anterior surface of the head are assumed to function as visual courtship signals during head rocking [28].

*Bactrocera* males performing anal beating/fanning during the aggregation phase continue to perform this behavior during the courtship phase. In contrast, *B. oleae* males start to stridulate only when they have detected a female. They produce an intermittent sound when averted from the female and a continuous sound lasting one to two seconds when facing the female [46].

Tactile stimuli during courtship have only been observed in *C. capitata*: during the second stage of head rocking, the male used his aristae (antennae) to repeatedly tap the female [47].

##### Copulatory Phase

The copulatory phase starts after the male has leapt on the female and copulates. This phase was intensively investigated in *C. capitata*. The male usually leaps from a face to face position on the dorsum of the female, briefly buzzing with his wings (“wing buzzing”) and rocking his body rapidly forward and backward (“body rocking”). Subsequently, the male turns around towards the female head and attempts to copulate. If the female does not evert her aculeus, the male repeatedly nips on the posterior edge of her eversible membrane with the posterior lobes of his lateral surstyli (“surstyli nipping”). He also crosses the hind legs under the posterior portion of the female’s abdomen and raises her oviscape (“aculeus rising”). Furthermore, he rubs her abdomen periodically with his hind tarsi and tibiae in an attempt to stimulate her to raise her abdomen (“hind tarsi rubbing”) [48]. These movements can be interpreted as copulatory courtship and may serve to induce as yet undetermined favorable post-intromission female responses [28].

In *Bactrocera*, some post-mount behaviors were also described. Rolli [46] found that *B. oleae* males flapped with their wings and produced a sound for a few seconds. Poramarcom [49] observed flapping with the wings during mounting of *B. dorsalis* and interpreted this behavior as a way to balance the male rather than being an acoustical signal. Body rocking and hind tarsi rubbing (“leg scrubs”) were observed in *B. oleae* [44].

In *A. rufipes*, post-mount behaviors were not observed. This may be due to the low number of observations (*n* = 2), fast movements of the flies and lack of high speed video recordings. In *A. rufipes*, the copulation lasted about 90 min or longer, in *C. capitata* about 90 to 195 min [28] and in *B. tryoni* about 35 min [37].

#### 4.2.2. Comparison of the Reproductive Behaviors of *Anoplomus*, *Ceratitis* and *Bactrocera*

The compilation in Table 2 shows that Dacinae used pheromones as long-distance signals (aggregation phase), pheromone, sound and visual stimuli as short-range signals (courtship phase) and tactile signals as contact-range signals (mainly copulatory phase).

Pheromones were emitted during courtship in all three tribes of Dacinae. *A. rufipes* and *C. capitata* elevated their abdomen, everted their anal pouch, distended their pleurae and also performed anal dabbing. In *Bactrocera* species, abdominal elevation, abdominal pleural distension and anal touching were lacking, but they exhibited the unique anal beating/fanning behavior.

Sound production occurred in both *Ceratitis* and *Bactrocera* during courtship. In *A. rufipes*, sound production did not occur during courtship, but sporadic wing vibration was observed during aggressive interactions between males in the laboratory.

Visual signals were more important in *A. rufipes* (wing displays, looping flights, lofting/body swaying movements), than in *Ceratitis* (simple head rocking behavior) or *Bactrocera* (visual stimuli not apparent). The importance of vision can also be seen from the fact that *A. rufipes* reacted to moving objects at a distance of *ca*. 30 cm or more, *Ceratitis* at a distance of probably less than 15 cm [18] and *B. cucurbitae* at a distance of about 15 cm [50].

The lofting/body swaying behavior of *A. rufipes* was performed during courtship, as well as during aggressive interactions. However, during the courtship observations in the field, lofting/body swaying was used only in the context of courtship behavior. In a few cases, males approached other males or other insects performing lofting/body swaying behavior, but they always interrupted the movements as soon as they recognized that the approached insect was not a female. Thus, it appears that during the courtship phase, the lofting/body swaying behavior is used exclusively as a courtship signal and not as a threatening gesture. Lofting or body swaying was also observed in males of Tephritinae as part of the courtship ritual or as a threatening gesture [11]. Looping flights have only been observed in two tephritid species of the Neotropical genus *Anastrepha* and occurred during courtship [14,51].

Tactile behaviors were manifold in *C. capitata* during the copulatory phase, while in *Bactrocera*, they seemed to be less pronounced or have been examined less intensively. In *A. rufipes*, detailed observations are lacking.

In summary, the comparison of courtship behaviors of the three genera shows that *A. rufipes* is more similar to *C. capitata* than to *Bactrocera* spp., especially with regard to the pheromone calling behavior and anal dabbing. Visual stimuli played an important role during the courtship phase in *A. rufipes*, while in *C. capitata* and *Bactrocera* spp., sound production was more pronounced.

#### 4.2.3. Choice of the Mating Site

*A. rufipes* individuals mated at the beginning of the rainy season on the upper surface of bamboo leaves of *P. albociliata*. Bamboo shoots (oviposition sites) were not available at this time of the year.

The growth of *P. albociliata* from bamboo rhizomes was caused by fires, which had burnt down the vegetation during the hot season. The young bamboo plants provided food (exudates of extrafloral nectaries) especially for the *A. rufipes* females. In areas not affected by fire, bamboo growth was negligible, and extrafloral nectaries or *A. rufipes* individuals were not present. At the end of the observation period, *A. rufipes* abandoned the site, probably because the development of the bamboo branches was completed and the bamboo nectaries had ceased to function.

In the following year (2015), the same study site was regularly visited between April and July. In 2015, the area was not affected by fire; bamboo growth was negligible and *A. rufipes* individuals were not present. All of these findings suggest that *A. rufipes* gathered at the mating site because of the high concentration of bamboo nectaries.

Additional factors playing a role in the choice of the mating site were the microclimatic conditions and structural characteristics of the vegetation. The flies always stayed in shady areas below large trees, and courtship displays were performed at the edges of the bamboo undergrowth stand, where vegetation was sparse, thus providing good sites for scanning the surroundings, performing looping flights and dispersing pheromones.

#### 4.2.4. Lek Formation

*Anoplomus rufipes* leks were established in the same areas where females were feeding and where individual males were searching for females. The males participating in a lek behaved in the same manner as single males. Thus, it is possible that the males met randomly in an area providing food and favorable microclimatic conditions and subsequently formed a lek.

The leks were rather ephemeral structures, and their cohesion was perhaps maintained by two contradictory forces, the attraction of males to each other and repulsion through aggressive interactions. Data on the mutual attraction of *A. rufipes* males are not available, but males of *C. capitata* release a pheromone that apparently attracts both males and females ([52,53], but see [54]). This is also true in other lek-forming tephritids like *Anastrepha suspensa* (Loew) [55] or in the lek-forming Drosophilidae [56]. Bioassays indicate that male-produced *Bactrocera* pheromone is effective from a distance of several meters, at least towards females [55]. Thus, pheromones of *A. rufipes* could attract other males and lead to the formation of a lek, while their agonistic interactions could result in the maintenance of a minimum distance between the males, which should be larger than the visual field of *A. rufipes*.

Pheromones emitted during calling do not explain why leks were repeatedly established at the same corner of the main study site for many consecutive days, although the area was relatively large and uniform. Scent marking, *i.e*., pheromones deposited on leaves during anal dabbing, could provide an explanation for this phenomenon.

The males of *A. rufipes* placed their scent markings during pheromone calling and then proceeded to neighboring leaves. This suggests that the scent markings rather act as a long-term attractant. It is known that during anal dabbing, *Anastrepha suspensa* deposits pheromones on leaf surfaces [13] and that some of the components remain there at least until the following day [2]. In both *A. suspensa* and *C. capitata*, leaves previously exposed to males were more attractive for mature virgin females than untreated leaves [13,54]. If it is assumed that the scent markings of *A. rufipes* are operative until the next day, the males could return to the marked leaves and place additional scent markings in the same area, thus enforcing the effect of the pheromones, as long as they benefited from staying at that specific location.

Scent marking could also facilitate the search for mates. Females were sometimes seen to move quickly in a straight line from leaf to leaf, dab the leaf surface and observe the surroundings from the edge of the leaf. It is likely that these individuals were searching for mates rather than food, since there were a lot of extrafloral nectaries available in the vicinity of the females. Scent markings could arrest searching females in areas of high courtship frequency, even if the male was not present in the immediate vicinity. If several males deposited scent markings, the marked area would be larger, making it easier for the females to find a lek area.

The number of *Anoplomus*, *Ceratitis* or *Bactrocera* individuals participating in leks was in a similar range. In *A. rufipes*, there were up to four males, in *C. capitata* three to four males under natural conditions and eight to sixteen following releases of mass-reared flies [18,28,57], in *B. cucurbitae* up to seven males [50] and in *B. cacuminata*, 10 to 15 calling males [43]. However, there were differences in the process of lek formation, the territorial behavior and the occurrence of scent marking.

The males of *A. rufipes* and *C. capitata* constantly changed their positions between leaves and did not defend a certain leaf territory, but rather kept other males at a distance. Attacking males of *A. rufipes* were often successful in evicting opponents, and field-observed males of *C. capitata* also won significantly more combats than resident males [57]. Further similarities were that *C. capitata* performed anal dabbing, that leks were stable over time at the same locations and that males also searched for females individually [28,57].

In contrast, males of *B. cacuminata* and other *Bactrocera* species formed a loose swarm between the leaves, which shifted to different parts of the tree. The swarm then settled in one part of the tree while some males set up single-leaf territories. Intruding males of *B. cacuminata* and *B. oleae* were usually evicted by the resident male [43,58]. Anal dabbing was not observed in *Bactrocera*, but according to the description of anal beating/fanning by Kuba and Sokei [35], it is possible that pheromones were sprayed on the leaf surface during this behavior. Kuba and Koyama [59] suspected that the sex pheromone of *B. cucurbitae* remained around mated pairs, because females approached them and behaved as if they were males. However, the effect was visible only for a short time.

There are many hypotheses proposed for the evolution of insect leks, for example that leks amplify the male signals, facilitate female choice or that leks are just simple accumulations of calling males in favorable microhabitats [2].

In the present study, it is hypothesized that individual *A. rufipes* males searching for females meet randomly in areas providing favorable microclimatic conditions and food. Scent markings deposited on leaves are maybe employed as long-term attractants in order to arrest searching females and males at a particular location and could also account for the persistence of leks at the same location over a number of days. The function of lek formation is probably to concentrate males and females in a small area, get the flies in a state of higher excitation and thus maximize the encounter rates between males and females.

#### 4.2.5. Mating System in Gastrozonini

Distributions of breeding sites, food or other resources influence where the sexes meet [60]. When female-required resources are relatively rare and distributed in distinct patches, the male can occupy these resources, only allowing access in return for copulation (resource defense polygyny [60] or resource-based mating system [1,61]). Therefore, there is no need for males to invest in displays, and there is little or no courtship to be expected.

When resources are abundant and homogenous, waiting for females is unlikely a profitable method for encountering mates. Instead, the males will search for females or attract them by producing long-distance signals or establishing leks. Females have the choice to leave, and therefore, the males are forced to invest in elaborate courtship displays (non-resource-based mating system).

Males and females of *A. rufipes* met in areas of high concentrations of bamboo nectaries, which were evenly distributed. Under these conditions, the males adopted a searching strategy, performed pheromone calling and established leks. Thus, *Anoplomus* has a non-resource-based mating system like many frugivorous lek-forming species of *Bactrocera*, *Ceratitis* or *Anastrepha*.

Females of species having non-resource-based mating systems, for example *C. capitata*, *Bactrocera* or *Anastrepha* spp., mate one or a few times [14,28,62]. The frequency of matings in *A. rufipes* is also expected to be low, because multiple matings are rather found in species that exhibit resource defense mating systems [11]. However, multiple matings were observed in the Gastrozonini *Enicoptera gigantea* [10], indicating that this or other Gastrozonini may have a resource defense mating system.

## 5. Conclusions

The mating system of the non-frugivorous *A. rufipes* (Gastrozonini) is similar to the mating systems found in the frugivorous lek-forming species of *Ceratitis* (Ceratitidini) and *Bactrocera* (Dacini). Comparisons of the reproductive behaviors and lek formation suggest that the Gastrozonini are more closely related to the Ceratitidini than to Dacini. Further research on the reproductive behavior in tephritids should attempt to increase our basic knowledge of the response of Dacinae to pheromones or visual cues. Comparative behavioral and ecological studies of Dacinae (more than 800 species) could contribute to our knowledge of the functioning of mating systems and of the phylogeny of this group.
